# Efficacy of Capecitabine and Temozolomide in Small Bowel (Midgut) Neuroendocrine Tumors

**DOI:** 10.3390/curroncol29020046

**Published:** 2022-01-26

**Authors:** Taymeyah Al-Toubah, Brian Morse, Jonathan Strosberg

**Affiliations:** 1 Department of GI Oncology, H. Lee Moffitt Cancer Center and Research Institute, Tampa, FL 33612, USA; Taymeyah.al-toubah@moffitt.org; 2 Department of Diagnostic Imaging, H. Lee Moffitt Cancer Center and Research Institute, Tampa, FL 33612, USA; brian.morse@moffitt.org

**Keywords:** neuroendocrine tumors, capecitabine, temozolomide, captem, small bowel, carcinoid, midgut

## Abstract

**Simple Summary:**

The capecitabine/temozolomide (CAPTEM) regimen has proven activity in pancreatic neuroendocrine tumors (NETs); however, data are limited in NETs of the small bowel. To observe whether this regimen has activity in this patient population, we conducted a retrospective study of all patients with small bowel NETs treated with this regimen at our institution. We found that response rates were poor among patients with low-intermediate grade tumors but higher among patients with high-grade disease. Our findings suggest that the CAPTEM regimen should be reserved for patients with higher-grade small bowel NETs.

**Abstract:**

The capecitabine/temozolomide regimen has significant activity in pancreatic NETs; however, data are limited in NETs of the small bowel (midgut). A retrospective study of all patients with metastatic midgut NETs seen at Moffitt Cancer Center between January 2008 and June 2019 treated with CAPTEM was conducted. 32 patients with proven or suspected well-differentiated primary small bowel NETs (excluding duodenum) were identified. 6 patients were found to have a radiographic response (19%), 5 of whom had high-grade disease. Only one patient among 23 with low/intermediate-grade disease responded (4%), whereas the response rate for patients with high-grade disease was 56%. Among patients with low/intermediate-grade disease, 44% discontinued due to poor tolerability. The CAPTEM regimen appears to have an activity in patients with high-grade small bowel NETs and is largely inactive in patients with low/intermediate-grade tumors.

## 1. Introduction

Gastroenteropancreatic neuroendocrine tumors (GEP-NETs) are clinically and biologically heterogeneous neoplasms [1]. The two most common subtypes, midgut and pancreatic NETs, are distinct in their underlying mutational landscape, hormonal secretion profile, and response to various anti-cancer therapies [2,3]. Midgut NETs, originating in the jejunum and ileocecum, are almost invariably malignant but generally slow-growing. Pancreatic NETs are more equally distributed between indolent and aggressive and are characterized by higher objective response rates to most therapies, including targeted drugs and peptide receptor radiotherapy (PRRT) [4,5]. One of the most striking differences between pancreatic and midgut NETs is their differential response to cytotoxic therapy, with objective response rates (ORR) of approximately 50% reported in patients with pancreatic NETs treated with alkylating or platinum-based regimens, versus 0–10% among midgut NET patients [6,7,8,9,10].

In recent years, the most studied cytotoxic regimen in the NET field has consisted of capecitabine and temozolomide (CAPTEM). ORRs of 30–70% have been reported in single-armed studies of pancreatic NETs [8,11,12,13,14,15,16]. In addition, the randomized phase II ECOG 2211 trial demonstrated a significant improvement in progression-free survival (PFS) and overall survival (OS) with the CAPTEM combination compared with temozolomide monotherapy [14]. As a result, CAPTEM is now recommended by multiple expert consensus guidelines for the treatment of advanced, progressive pancreatic NETs [17,18,19].

While data on the efficacy of CAPTEM in midgut NETs are largely absent, studies of other temozolomide combinations, such as temozolomide/thalidomide and temozolomide/bevacizumab, have demonstrated negligible response rates in gastrointestinal NETs [20,21]. One recent study of CAPTEM in GEP-NET patients with ki-67% < 55% included four patients with small bowel NETs; however, the outcomes of this specific population are not reported [22]. As a result, cytotoxic chemotherapy, including CAPTEM, is generally not recommended for use in the midgut NET patient population. Indeed, cytotoxic drugs are listed as category III (no consensus/uncertain benefit) in the National Comprehensive Cancer Network (NCCN) guidelines [17].

Several studies have explored the association between the expression of the DNA repair enzyme methyl-guanine-methyltransferase (MGMT) and response to temozolomide-based chemotherapy. However, the results of these analyses have been inconclusive, and the analysis of MGMT is not currently recommended by guidelines or routinely performed [8,14,23,24,25].

Tumor kinetics may represent another resistance factor with a minimal proliferative threshold required for response to chemotherapy. We hypothesized that well-differentiated, high-grade midgut NETs, defined by a mitotic rate or ki-67 proliferative index >20%, may be uniquely sensitive to cytotoxic chemotherapy compared to lower grade midgut NETs. To address this hypothesis, we reviewed our experience with CAPTEM in midgut NETs, with a focus on the comparison of high-grade tumors versus low/intermediate-grade disease.

## 2. Materials and Methods

The records of all patients with small bowel (jejunal/ileal) midgut NETs treated with CAPTEM and seen at the Moffitt Cancer Center between January 2008 and June 2019 were reviewed. Institutional review board approval was acquired, and waiver of consent was obtained due to the study’s retrospective nature. Patients who were prescribed CAPTEM at outside institutions at appropriate doses and schedules were included in this analysis. Duodenal NETs were not included in this analysis.

The primary endpoint of this study was ORR by RECIST 1.1, stratified by tumor grade (high grade versus low/intermediate grade). Baseline scans and scans showing maximal response were reviewed by a radiologist (B.M.) to confirm the percent of tumor shrinkage. Progression-free survival (PFS) was a secondary endpoint and extrapolated by reviewing scan reports and clinical notes. Other endpoints included overall survival (OS) and toxicity. Data were analyzed using IBM SPSS version 26. Survival curves were estimated using the Kaplan–Meier methodology, and categorical variables were analyzed using logistic regression. A *p*-value of 0.05 was used for Pearson correlations and chi2 analyses.

## 3. Results

Within a database of 462 patients with metastatic NETs treated with CAPTEM, 32 patients with proven or suspected primary small bowel NETs (excluding duodenum) were identified. Twenty-three patients had low- or intermediate-grade tumors, and nine had high-grade well-differentiated (G3) NETs with ki-67 indexes ranging from 22–80%. All patients had received somatostatin analogs (octreotide or lanreotide), except for one who was initially diagnosed as a pancreatic NET with the diagnosis later corrected, eight patients had prior surgery (two liver debulking, seven resections of primary tumors), 12 patients had received liver embolization, four patients had prior peptide receptor radiotherapy (PRRT), and five patients had received everolimus. Table 1 describes the patient demographics.

The median duration of treatment was eight months (range: 0–73); five months for patients with low/intermediate-grade tumors and 22 months for patients with high-grade tumors. At the time of the data cut-off, two patients remain on active treatment (35 and 48 months of treatment to date), both of whom have high-grade disease.

Partial radiographic response (PR) was achieved in six patients, five of whom had high-grade (well-differentiated) disease. Only one patient among 23 with low/intermediate-grade disease responded (4%), whereas 5/9 patients (56%) with high-grade disease responded to CAPTEM therapy (*p* < 0.001).

**Figure 1:**
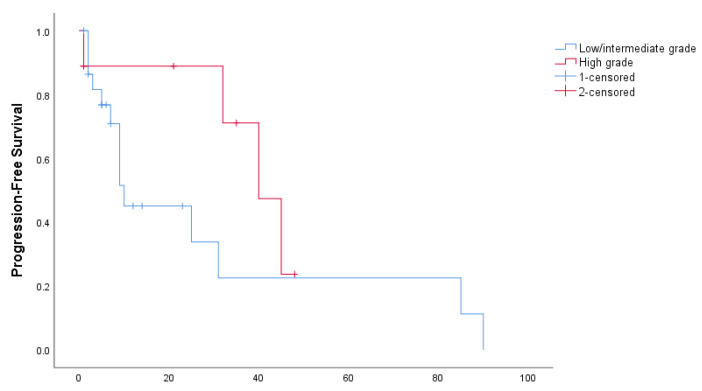
Progression-free survival, stratified by tumor grade.

**Figure 2:**
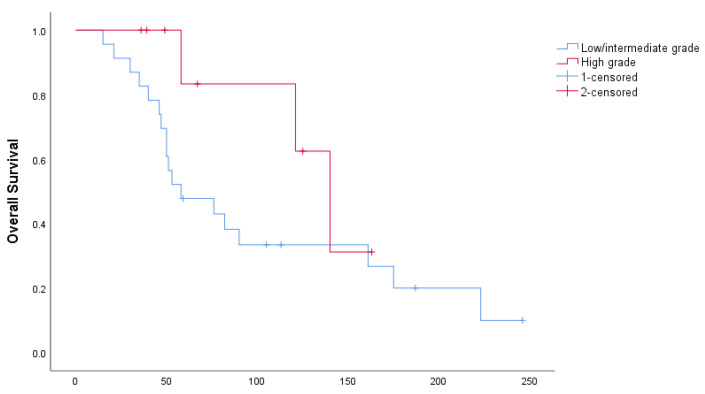
Overall survival, stratified by tumor grade.

Median PFS in the entire cohort of patients was 31 months (95% CI: 0–66.8 months): 10 months in patients with low/intermediate-grade tumors and 40 in patients with high-grade tumors (*p* = 0.176), as shown in Figure 1. Median OS for the entire cohort was 82 months (95% CI: 32.8–131.2 months): 58 months for low/intermediate and 140 months for high-grade (*p* = 0.093), as shown in Figure 2.

Among patients with low/intermediate-grade disease, 44% discontinued due to poor tolerability. Three patients (33%) with high-grade disease discontinued treatment due to poor tolerability. Side effects included pancytopenia’s (grade 3–4 in six patients), fatigue, nausea, vomiting and diarrhea.

## 4. Discussion

Temozolomide-based cytotoxic regimens are associated with substantially higher response rates in patients with advanced pancreatic NETs than in those with small bowel NETs, which are considered chemoresistant. Our retrospective study confirms that objective radiographic responses to CAPTEM are exceedingly rare in low/intermediate-grade small bowel NETs. However, high-grade (G3) small bowel NETs appear to be an exception. Well-differentiated G3 NETs have only recently been recognized as distinct entities from low/intermediate NETs and poorly differentiated neuroendocrine carcinomas (NEC) [26]. In addition, G3 NETs represent a small minority of gastroenteropancreatic NETs and an even slimmer minority of small bowel NETs. Our data suggest that CAPTEM should be considered a treatment option in this rare population of patients.

Tumor kinetics likely explain the differential response of G3 small bowel NETs to cytotoxic drugs. Metastatic small bowel NETs are most commonly low-grade and slowly progressive. Although alkylating agents such as temozolomide are not considered cell-cycle-specific, cytotoxicity does depend on DNA replication. Antimetabolites such as capecitabine may be even more dependent on cell division for activity. Indeed, other studies have demonstrated a correlation between the tumor grade and objective response, including one which demonstrated that a ki-67 range of 10–40% was associated with the highest response rates [27].

It is well-established that the tumor grade can increase over time, either spontaneously or potentially in response to treatment. CAPTEM can be considered in patients with low/intermediate-grade small bowel NETs who develop rapidly progressive tumors after prior lines of treatment. Some guidelines advocate a repeat biopsy in cases of suspected grade transformation, although the necessity of this is debatable.

Limitations of our study include the small sample size and a particularly small subset of patients with G3 disease. The unusually long median PFS and OS we observed in the high-grade cohort are almost certainly overestimates, reflecting the low number of progression and death events. We did not evaluate any of the patients in this manuscript for MGMT expression. Prospective trials focusing on this particularly rare subpopulation are unlikely to be developed. In addition, the impact of CAPTEM on PFS is challenging to assess in the absence of a randomized controlled study. Thus far, the only randomized study of CAPTEM has been completed in pancreatic NETs, demonstrating a significant PFS and OS benefit. Similar studies in nonpancreatic NETs have not been developed.

## 5. Conclusions

In conclusion, CAPTEM is associated with meager rates of objective response in patients with metastatic small bowel NETs except for high-grade (G3) NETs. The use of CAPTEM in this population should be reserved for patients with aggressive disease.

## Figures and Tables

**Table 1:** Patient demographics and tumor characteristics.

	n, %	
Gender		
Male	17	53.1%
Female	15	46.9%
Age, years		
25–44	3	9.4%
45–60	9	28.1%
61–74	18	56.3%
75+	2	6.2%
# of Prior Lines		
0	1	3.1%
1	17	53.2%
2	8	25%
3	4	12.5%
4	1	3.1%
5	1	3.1%
Ki67 proliferation index		
≤2%	3	9.4%
3–20%	6	18.8%
21–80%	9	28.0%
Unknown	14	43.8%

## Data Availability

Data available upon request.
